# Biliary Rhabdomyosarcoma in Pediatric Patients: A Systematic Review and Meta-Analysis of Individual Patient Data

**DOI:** 10.3389/fonc.2021.701400

**Published:** 2021-09-30

**Authors:** Juri Fuchs, Anastasia Murtha-Lemekhova, Markus Kessler, Patrick Günther, Alexander Fichtner, Jan Pfeiffenberger, Pascal Probst, Katrin Hoffmann

**Affiliations:** ^1^ Department of General, Visceral and Transplantation Surgery, University Hospital Heidelberg, Heidelberg, Germany; ^2^ Department of General, Visceral and Transplantation Surgery, Division of Pediatric Surgery, University Hospital Heidelberg, Heidelberg, Germany; ^3^ Department of Pediatrics I, Division of Pediatric Gastroenterology, University Children’s Hospital Heidelberg, Heidelberg, Germany; ^4^ Department of Gastroenterology and Hepatology, University Hospital Heidelberg, Heidelberg, Germany; ^5^ Study Center of the German Surgical Society (SDGC), University of Heidelberg, Heidelberg, Germany

**Keywords:** biliary rhabdomyosarcoma, pediatric rhabdomyosarcoma, pediatric liver tumors, RELIVE initiative, pediatric oncology, pediatric liver surgery, pediatric hepatobiliary surgery

## Abstract

**Background:**

The biliary tree is a rare location of pediatric rhabdomyosarcoma. Due to the low incidence, there is a lack of evidence concerning therapeutic guidelines for this tumor location. In particular, the impact of surgery is discussed controversially.

**Purpose:**

Objective is to generate evidence-based treatment guidelines for pediatric biliary rhabdomyosarcoma (BRMS). All available published data on therapeutic regimens and important prognostic factors are investigated with a focus on the role of surgery.

**Methods:**

A systematic literature search of MEDLINE, Web of Science, and CENTRAL was performed. Patient data were entered individually. Data was pooled and qualitative and quantitative analyses of demographic data, therapy, postoperative/interventional outcomes, relapse, and survival were conducted. In an individual patient data analysis, cox regression was applied to identify key factors predicting the outcome of patients with BRMS.

**Results:**

65 studies met the inclusion criteria, providing data on 176 patients with BRMS. Individual patient data analysis showed a 5-year overall survival and progression-free survival of 51% and 50% for the total study population. For patients treated after 2000, 5-year OS and PFS was 65% and 59%, respectively. Absence of surgical tumor resection was an independent risk factor for death (Hazard ratio 8.9, 95%-CI 1.8-43.6, *p* = 0.007) and significantly associated with recurrent disease and disease-related death.

**Conclusion:**

This analysis provides comprehensive information on the largest number of patients hitherto reported in the literature. BRMS is still associated with high morbidity and mortality. Surgical tumor resection is essential for appropriate oncological treatment of BRMS. International cooperation studies are needed to enhance evidence and improve the outcome of this orphan disease.

**Protocol Registration:**

PROSPERO (CRD42021228911) https://www.crd.york.ac.uk/prospero/display_record.php?ID=CRD42021228911.

## Highlights

➢Not performing surgical tumor resection increases the risk of relapse and death in BRMS.
➢Delayed surgery achieves higher rates of microscopical complete tumor resection compared to upfront surgery.
➢BRMS in children is still associated with considerable mortality.
➢Relapse of BRMS has mostly fatal outcome.
➢Alveolar histology is rare but possible in BRMS and is associated with poor survival.
➢Further research is needed for the development of the best multi-modal treatment strategy to improve outcomes and avoid long-term sequelae in children with BRMS.

## Introduction

Primary tumor location of rhabdomyosarcoma (RMS) in the biliary tract accounts for just 0.5 to 1.5% of all RMS in children (1–4). Nevertheless, biliary rhabdomyosarcoma (BRMS) is the most common malignant cause of obstructive jaundice in pediatric patients. Tumors can arise from anywhere in the extra- and intrahepatic biliary tract, with the common bile duct being the most frequent primary tumor site (1–5).

Given the rarity of this disease, there is a lack of evidence-based diagnostic and therapeutic recommendations. Many therapeutic principles are based on oncological studies comprising a broad variety of RMS types in children. For patients affected by particular subtypes, such as BRMS, these unspecific and wide-ranged recommendations may lead to poorer treatment. This is reflected by the lack of substantial improvements in the therapy for high-risk and relapsed RMS patients (6, 7). For example, the biliary tract had been classified as “*favorable site*” in recent Children’s Oncology Group (COG) studies (1), meaning that this primary tumor location is assumed to have a better prognosis, thus requiring a less aggressive therapy (8). This decision was based on positive outcomes of 25 patients treated for BRMS over a period of 26 years (5) – a scientific data basis that seems sparse to be accepted for treatment recommendations. The classification as favorable site has been questioned due to a lower 5-year OS of children with localized BRMS of 76.5% compared to the expected 85%, when treating localized BRMS in low-risk trials (1).

The literature on BRMS is scarce with predominantly case reports and small case series available. Only few retrospective studies from oncological trial registries selectively analyzed BRMS, reporting on samples sizes between 10 and 30 patients (1–5, 9). Several vital questions concerning the best suited therapy according to age, tumor location, size and stage remain unanswered. In particular, the impact of surgery has caused controversies in recent years. While the resection of bile ducts, including extended surgery with liver resection and vascular reconstruction in the porta hepatis, have become standard procedures in oncologic hepatobiliary surgery for adults (10, 11), extended surgery in pediatric patients is often questioned and some oncologic studies cast doubt over the benefit of surgical resection in BRMS at all (1, 5). This scepsis may be based on the observation of complete remission in some patients with BRMS after sole chemotherapeutic treatment. In contrast, other authors emphasized the advantages of complete surgical tumor resection in pediatric RMS (12, 13) and the need for local therapies for BRMS even in complete remission after chemotherapy (3). However, reliable evidence is missing.

Within the framework of the interdisciplinary *RELIVE Initiative* (Position paper to be published soon), the aim of this systematic review with meta-analysis of individual patient data is to generate evidence-based recommendations for an improved and individualized therapy of BRMS by synthesizing all available data. The focus of this analysis is to clarify the impact of surgery on the outcome of patients with BRMS.

## Materials and Methods

### Review Structure, Ethics and Search Strategy

This review was conducted in accordance with the *Preferred Reporting Items for Systematic Reviews and Meta-Analyses for Individual Patient Data (PRISMA-IPD)* guidelines (14). Before starting the literature search, the methods were predefined. The project was registered with the International *Prospective Register of Systematic Reviews* (PROSPERO) prior to the study selection (PROSPERO 2021 CRD42021228911). In all included studies, ethical approval from the relevant committees was reported. Only anonymized data were investigated in our analyses. Thus, the institutional review board of the Medical Faculty of the University of Heidelberg approved the data collection and conduct of the present study (Sign 07/2013) and no additional patient consent was necessary (section 15, paragraph 1 of the code of medical ethics of the federal state of Baden-Württemberg, Germany).

To retrieve all articles reporting on pediatric patients with the diagnosis of RMS arising in the biliary tract, *MEDLINE* (via PubMed), *Web of Science*, and *CENTRAL* were searched using a combination of the following medical subject heading (MeSH) terms and free text terms: *biliary, hepatobiliary, botryoides, botryoid, bile duct, rhabdomyosarcoma, sarcoma, bile duct neoplasms, biliary tract neoplasms, child, infant, pediatric, adolescent.* The complete search strategy is provided in the **Supplementary Material (File 7)**. Additionally, reference lists of the relevant literature were searched for eligible studies. No language restrictions were defined. Only studies published in 1950 or later were eligible – earlier reports were regarded as unsuited for comparison due to the lack of clear histological definitions of RMS and, thus, insufficient differentiation from other types of sarcoma, and due to the absence of comparable therapeutic measures such as chemo- and radiotherapy in the treatment of oncologic patients at the time. The last search was conducted on March 20^th^, 2021.

### Study Selection Criteria and Selection Process

Studies of any methodology that included pediatric patients with biliary tract rhabdomyosarcoma were regarded as eligible. The following inclusion criteria were defined: documented histologic diagnosis of rhabdomyosarcoma with primary tumor location in the biliary tract, patient age ≤ 18 years, information on applied therapy and patient outcome available. In some studies, no information on therapy and/or patient outcome was provided, or only aggregated patient data were reported. Studies with missing information were excluded. In case of unclear information, clearing up was requested from the corresponding author of these studies. Studies that allowed only partial access to IPD were included for pooled data statistics but excluded from the IPD analysis. All abstracts of studies retrieved by the systematic literature search were screened for eligibility according to the above-mentioned criteria by two reviewers (JF and AML) independently. Afterwards, the full texts of all eligible articles were assessed for inclusion by JF and AML independently. Dissent between the two reviewers was resolved after discussion with a third reviewer (KH).

### Data Extraction and Investigated Variables

Before the data extraction, a standardized form was compiled. A full list of all extracted variables is added as **Supplementary Information 4**. The extracted variables were predefined based on relevant literature on pediatric (biliary tract) rhabdomyosarcoma, pediatric oncology, and pediatric hepatobiliary surgery. The two reviewers (JF and AML) independently extracted the data according to this form.

### Risk of Bias Assessment

Due to the rarity of the diagnosis, predominantly case reports/series and retrospective registry publications were anticipated. For all case reports and series, Murad et al.’s tool for risk of bias assessment was applied (15). According to this method, the risk is rated in four domains: “Selection”, “Ascertainment”, “Causality” and “Reporting”. For each domain, the risk of bias is classified as either “low”, “moderate” or “high”. An overall judgement is inferred after assessing each individual domain (15). For observational studies, MINORS was used (16). For non-randomized, non-comparative studies, as represented in this systematic review, this tool includes eight items, each being assessed and scored with either 0 (not reported), 1 (reported but inadequate) or 2 (reported and adequate) points (16).

### Statistical Analyses and Certainty of Evidence

All statistical analyses were performed using R (version 3.6.2.) (17), including the *survminer* (18) and *survival* (19) packages for survival curves and the *forestmodel* (20) package for forest plots. Data from all studies were pooled and descriptive statistics were calculated: means or medians with standard deviations (SD) or, for variables containing outliers, interquartile range (IQR) or range are given for continuous data; numbers with percentages are presented for categorial data. Univariate analyses were performed using the chi-squared test (without Yate’s correction) and the Mann-Whitney U test at a level of significance of 2.5%. Available IPD were pooled and 5-year overall survival (OS) and 5-year progression-free survival (PFS) were calculated using the Kaplan-Meier estimator with right censoring. OS was defined as time from diagnosis to death. PFS was defined as time between diagnosis and progression of disease, relapse, or death. Univariable significance of factors on (5-year-) OS and PFS was tested using the log rank test at a level of significance of 5%. The independent predictive value of different patient-related and treatment variables was analyzed applying a Cox regression model. By including all patient- and treatment-related covariates in the multivariable model, that showed significant association in univariable analyses (p < 0.05), possible biases were controlled for. The certainty of evidence and strength of recommendations was assessed according to the GRADE criteria (21).

## Applied Terms and Definitions

### Staging and IRS Classification (Resection Status)

Tumors were staged according to previously investigated risk factors in pediatric BRMS, i.e., tumor size (≤ 5 cm vs. > 5 cm), nodal involvement, and presence of distant metastases (3, 5). Patients were classified into clinical risk groups after initial surgery or biopsy using the Intergroup Rhabdomyosarcoma Study Group (IRS) guidelines (I: Complete surgical resection (R0); II: Microscopically incomplete surgical resection (R1); III: Gross tumor residual after surgery/biopsy (R2); IV: Metastatic disease) (22).

### Types of Surgery

Upfront surgery was defined as a surgical tumor resection before neoadjuvant (radio)chemotherapy. Delayed primary resection (DPR) was defined as surgical tumor resection after neoadjuvant chemotherapy, re-resection as tumor resection after initial upfront surgery, and chemotherapy. The following types of surgery were defined:

Limited (conservative) (3, 5) surgery:
(1) Local tumor excisions/enucleations (e.g., segmental excision of bile ducts, atypical liver resection)
(2) Resection of extrahepatic bile ducts (usually with Roux-en-Y reconstruction) without resection of the hepatic ducts or the pancreatic head or other neighboring organs
Extended surgery:
(1) Resection of extra- and partly intrahepatic bile ducts, with resection of parts of the hepatic ducts and/or liver resection
(2) Major liver resections (≥ 4 liver segments)
(3) Partial pancreaticoduodenectomy with resection of extrahepatic bile ducts
(4) Liver transplantation

If more than one surgery with intent of tumor resection was performed in one patient, the more invasive one was classified. Postoperative complications were classified according to the Clavien-Dindo Classification (23).

### Chemotherapy

Neoadjuvant chemotherapy (NAC) was defined as at least one course of cytostatic drugs before an attempted surgical tumor resection. In most cases, chemotherapy was then continued after surgery according to the respective study protocols. Adjuvant chemotherapy (AC) was defined as treatment that started after surgical tumor resection.

### Radiotherapy

The term radiotherapy was defined as external beam radiation therapy (EBRT) targeting the involved primary tumor field or areas of local lymph node metastasis. Patients who received radiotherapy with surgery were differentiated from those who were treated with definitive radiotherapy without surgical tumor resection.

### Outcome, Remission and Relapse

Disease-related death (DRD) was defined as death related to progression of the tumor, to postoperative complications or to adverse events of chemo- or radiotherapy. Complete remission was defined as no evidence of residual disease on imaging and/or at delayed explorative surgery after therapy. Partial remission was defined as primary tumor reduction and no evidence of new distant disease after therapy. Relapse was defined as recurrence of tumor (locally at the primary tumor site or distant metastasis) after either complete remission or progression of residual tumor and/or distant metastasis after partial remission. Disease progression during initial therapy was defined as tumor growth or absence of tumor reduction during first line treatment.

## Results

### Literature Search and Study Selection

**Figure 1:**
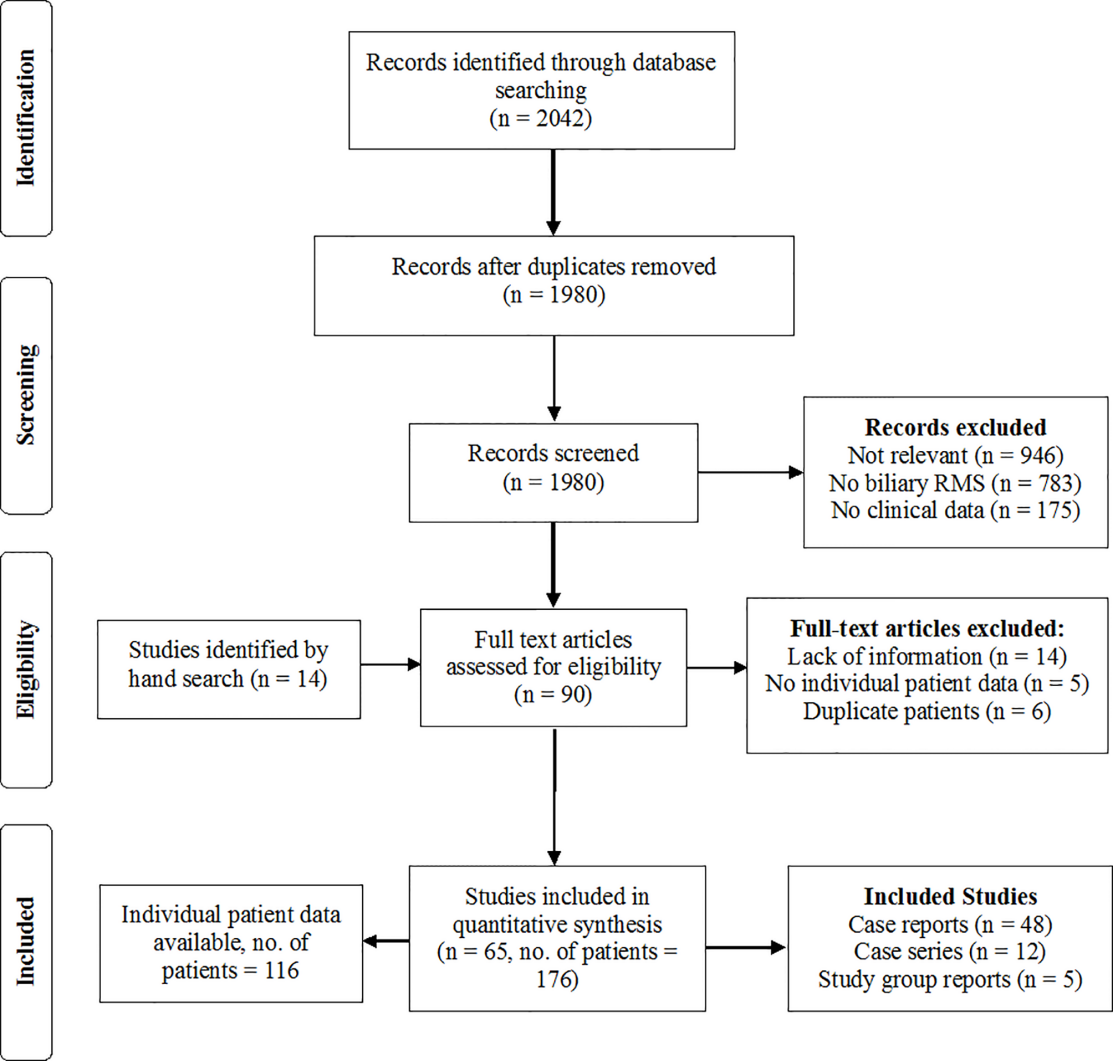
PRISMA flow chart of the study selection and inclusion process.

After removing duplicates, 1980 articles were retrieved by the systematic literature search. 76 full texts were assessed for eligibility. Additional 14 eligible case reports were found by screening the reference lists of the relevant literature and by hand search. In 35 of these 90 studies, there was not sufficient information on therapy and patient outcome, and most of these studies had to be excluded from the analysis. In case of doubt about the validity of the histologic diagnosis or the applied therapy and outcomes, the corresponding authors of these articles were contacted with the request for definite information. In five cases, the request was denied. In another 16 cases, the authors could not be traced, the missing information could not be retrieved by the authors, or the authors did not reply (List of excluded studies in **Supplementary Information 5**). Studies were included upon reception of sufficient information. Three studies that reported exclusively on patients with BRMS, but provided only limited access to IPD, were included for the pooled data analysis but were not suited for IPD analyses (1–3). In 11 cases, the same patient was reported by more than one case report or study. In these constellations, the source with more detailed data was included and other reports on the patient were excluded from the analysis to avoid double reporting. Ultimately, 65 studies were included in the analyses, consisting of 48 case reports, 12 case series and five reports from oncological study groups, providing data on 176 patients. **Figure 1** (List of included studies in **Supplementary Informations 1**).

### Critical Appraisal of Included Studies and Risk of Bias Assessment

As expected, none of the included studies was comparative and the majority were case reports. The risk of bias (RoB) of the 48 case reports and series was assessed using the above-mentioned and described tool by Murad et al. (15). The majority (42/60) of case reports were rated with a moderate or low risk of bias. Selection bias was high in most case reports, since mostly individual patients were reported. On the other hand, this implied that detailed description of patients, including adequate follow-up, was provided. This allowed us to perform an extensive IPD analysis and control for many variables. The mortality among all included cases was 35%, which is relatively high. This indicates that not only positive outcomes were reported and reveals a comparatively low publication bias in this regard. For the five observational studies, the *MINORS* tool for RoB was applied. The studies reached between 13 and 14 points, indicating a moderate to low risk of bias. All five studies present retrospective analyses of patients with BRMS, who were treated according to protocols of oncologic studies including pediatric patients with various types of RMS. Although these oncologic trials were prospective and included randomization of certain patient groups, there was no randomization comparing different local therapies within the subgroup of patients with BRMS. Selection bias was low in all five studies, as they included consecutively recruited patients in well-organized study registries (Tables with RoB for all studies in **Supplementary Informations 2, 3**).

### Pooled Data Analyses - Patient Characteristics

The included articles provided information for a total of 176 pediatric patients with BRMS. Median patient age at diagnosis was 36 months (IQR 30-52, range 4-204 months). Patients were divided into three groups depending on the time of treatment: 31 patients were treated between 1952 and 1980, 58 between 1981 and 1999, and 87 after the year 2000. Embryonal histology was predominant with 59%, followed by botryoid subtype in 39% of cases. Alveolar histology is very rare but possible in BRMS and was diagnosed in four children (2%), all of which died of disease (**Table 1**).

**Table 1:** Patient characteristics.

Total number of patients n = 176	
Median age at diagnosis	36 months (IQR 30-52, range 4 – 204 months)
Gender	male = 101 (57%); female = 75 (43%)
Treatment 1950 – 1980	31 (18%)
Treatment 1981 – 1999	58 (33%)
Treatment after 2000	87 (49%)
Treatment within study protocol	127 (72%)
Presenting symptoms	
Jaundice	108 (61%)
Abdominal pain	40 (23%)
Fever	36 (20%)
Loss of appetite/Anorexia	19 (11%)
Vomiting	12 (7%)
No information	46 (26%)
Median serum bilirubin (at diagnosis)	9.8 mg/dl (IQR 5.1-12.7)
Primary diagnostic methods	
Ultrasound	47 (26%)
CT scan	63 (35%)
MRI	58 (33%)
ERCP	12 (7%)
Other/No information	55 (31%)
Initially suspected diagnosis	
Choledochal cyst	29 (16%)
Malignant hepatobiliary tumor	41 (23%)
Infectious hepatitis	11 (6%)
Unclear solid tumor	7 (4%)
Parasitic disease	4 (2%)
Biliary rhabdomyosarcoma	2 (1%)
Other/No information	84 (48%)
Location of tumor origin	
Intrahepatic origin	26 (15%)
Extrahepatic origin	114 (65%)
Not specified	36 (20%)
Details of tumor location	
Common bile duct	68 (39%)
Hepatic ducts (extrahepatic)	7 (4%)
Cystic duct/Gallbladder	8 (5%)
Liver (intrahepatic bile ducts)	26 (15%)
Ampulla of Vater/Ampullary region	5 (3%)
No information on exact location	62 (35%)
Mean tumor size	7 cm (Range 2 – 30 cm)
Tumor stage	
Tumor ≤ 5cm	66 (38%)
Tumor > 5cm	109 (62%)
Tumor size unknown	1 (1%)
N0	91 (52%)
N1	27 (15%)
N status unknown	58 (33%)
M0	157 (89%)
M1	19 (11%)
Histology	
Embryonal	104 (59%)
Botryoid subtype	68 (39%)
Alveolar	4 (2%)
IRS Clinical Group	
I	19 (11%)
II	36 (20%)
III	102 (58%)
IV	19 (11%)

### Diagnostics

Information about diagnostic workup was heterogenous among the studies and missing in some cases. In recent years, magnetic resonance imaging and/or computed tomography have become integral parts of the diagnostic workup in cases of pediatric obstructive jaundice and tumor suspect. Additionally, endoscopic retrograde cholangiopancreatography (ERCP) has emerged as a diagnostic and possibly therapeutic modality in children with obstructive jaundice. It was applied in 12 cases in the presented cohort (7%, age range 1.6 – 8 years). Tumor biopsies were taken in these instances and allowed for correct definitive diagnosis, with one re-ERCP performed to establish correct histological results. One case of uncontrollable hemorrhage after endoscopic biopsy was reported, necessitating emergency surgery and tumor resection that resulted in long-term survival of the patient (24).

### Surgical Therapy and Local Treatment Regimen

#### Upfront Surgery

In 81 patients (46%) upfront surgery with intent of tumor resection was performed. Negative resection margins (R0 status, IRS Group I) were achieved in 19 of 81 cases (23%). In 17 of those 19 cases (89%) with R0 upfront resection, extended surgery was applied, which resulted in complete remission and long-term survival for 89% of these patients.

#### Delayed Surgery After Neoadjuvant Treatment

DPR was performed in 47 patients (27%). Tumor re-resection was performed in 6 patients (3%). In 35 of those 53 cases (60%), microscopically complete tumor resection (R0 status) was achieved. In 21 of those 35 cases with R0 resection margins and DPR, extended surgery was applied (60%). Localized surgery was performed in 7 (20%). The exact type of surgery was not specified for 7 of those patients (20%).

#### Type of Surgery

In the 128 patients with tumor resection, limited surgery was used in 60 cases (33%) and extended procedures were performed in 51 patients (29%). In 17 cases, the exact type of surgery was not specified. R0 status was confirmed in 52 cases (41% of all tumor resections, 30% of all patients), R1 in 49 (38% of all tumor resections, 28% of all patients) and R2 in 27 (21% of all tumor resections, 15% of all patients). R0 was significantly more often achieved with DPR compared to upfront resection (OR 6.3, 95%-CI 2.8-14.8, p < 0.001). Moreover, extended resections resulted significantly more often in R0 status than limited surgery (OR 17.8, 95-% CI 2.2-391.9, p < 0.001). There was no significant difference in the relapse and death rate between patients who underwent upfront resection versus those who received surgery after neoadjuvant treatment (DRD: 25% vs. 23%, *p* = 0.870, relapse: 27% vs. 26%, *p* = 0.961). 18 major postoperative complications (≥ Clavien-Dindo grade III) were reported in the 128 operations (14% of resections) and two postoperative deaths occurred (1%).

#### Surgery vs No Surgery

In 48 cases (27%), no surgical tumor resection was attempted. The mortality rate in this group was 63% (30 of 48 patients), and 21 patients relapsed after temporary (partial) remission (44%). The mortality rate in the group of 128 patients with surgical tumor resection was 24% (31 deaths). Based on univariate analysis, absence of surgical tumor resection was significantly associated with relapse and DRD (DRD: OR 5.2, 95%-CI 2.4-11.3, *p* < 0.001; relapse: OR 2.2, 95%-CI 1.1-4.8, *p* = 0.02).

#### Surgery With or Without EBRT

The mortality in patients with chemotherapy and surgical tumor resection without radiotherapy was 26% (15 of 57 patients), and 15 relapses occurred (28%). The mortality rate in the group treated with chemotherapy, surgical tumor resection and radiotherapy was 23% (16 of 71 patients), and 18 relapses occurred (25%). With regards to the outcome, there was no significant difference between the group with surgery plus EBRT and the group with surgery without EBRT (DRD: OR 1.2, 95%-CI 0.5-3.0, *p* = 0.62; relapse: OR 1.1, 95%-CI 0.4-2.5, *p* = 0.90) (**Tables 2**, **3**).

**Table 2:** Details of surgical therapy.

Total number of patients n = 176	
Upfront surgery*	83 (47%)
DPE or re-resection*	58 (33%)
Surgical tumor resection attempted*	128 (73%)
Limited (conservative) surgery*	60 (34%)
Extended surgery*	51 (29%)
Type of surgery not classified	17 (10%)
Type of surgical procedure^†^	
Explorative laparotomy with tumor biopsy	51 (29%)
Unspecified tumor resection	11 (6%)
Local tumor excision*	18 (10%)
Extrahepatic bile duct resection (limited)*	35 (20%)
Bile duct and hepatic duct resection (radical)*	23 (13%)
Major liver resection*	30 (17%)
Partial pancreaticoduodenectomy	11 (6%)
Liver transplantation	4 (2%)
Postoperative outcome	
Major postoperative complications*	18 (10%)
Postoperative death	2 (1%)
Re-Resections	14 (8%)
R-status	
R0	52 (30% of all patients, 41% of tumor resections)
R1	49 (28% of all patients, 38% of tumor resections)
R2 (without biopsies)	27 (15% of all patients, 21% of tumor resections)
R0 after upfront surgery (n=83)	19 (23%)
R0 after delayed or second surgery (n=58)	35 (60%)
R0 after limited surgery (n = 60)	13 (22%)
R0 after extended surgery (n = 51)	31 (61%)
R0 after undefined type of surgery (n = 17)	8 (47%)

*Definitions in the “Methods/Definitions” section. ^†^More than one procedure in one patient possible.

**Table 3:** Impact of local therapy on rate of relapse and DRD.

Surgery	Relapse [n]	OR*	95%-CI	p*	DRD [n]	OR*	95%-CI	p*
Surgical tumor resection: n = 128	33 (26%)	**5.2**	**2.4- 11.3**	**< 0.001**	31 (24%)	**2.2**	**1.1- 4.8**	**< 0.001**
No surgical tumor resection: n = 48	21 (44%)				30 (63%)			
Timing of Surgery								
Upfront surgery: n = 81	21 (27%)	1.0	0.4- 2.5	= 0.961	20 (25%)	1.1	0.4- 2.7	= 0.870
DPR (without re-resections): n = 47	12 (26%)				11 (23%)			
Surgery +/- EBRT								
Surgery without EBRT: n = 57	15 (28%)	1.1	0.4-2.5	= 0.901	15 (26%)	1.2	0.5- 3.0	= 0.620
Surgery with EBRT: n = 71	18 (25%)				16 (23%)			
EBRT								
Local EBRT: n = 93	24 (26%)	1.6	0.8- 3.3	= 0.138	25 (27%)	**2.2**	**1.1- 4.3**	**= 0.022**
No local EBRT: n = 83	30 (36%)				36 (43%)			
EBRT +/- Surgery								
EBRT without surgery: n = 22	6 (27%)	1.4	0.4- 4.7	= 0.857	9 (41%)	2.4	0.8-7.4	= 0.089
EBRT with surgery: n = 71	18 (25%)				16 (23%)			

*Chi-squared test. Bold values signify statistically significant results (p ≤ 0.05).

### Radiotherapy

External beam radiotherapy (EBRT) was applied in 93 patients (53%). Definitive radiochemotherapy without surgery was performed in 22 cases (13%). The mortality rate in this group was 41% (9 of 22 patients). EBRT combined with surgery and chemotherapy was applied in 71 patients (40%). 65 patients (37%) received adjuvant EBRT, 6 patients (3%) were treated with neoadjuvant EBRT. Median applied radiation dose was 41.4Gy (Range 5- 60Gy, SD 9.0). Median applied dose was significantly higher in patients who received RT without surgery compared to those who received EBRT with surgery (median 50.4Gy vs. 41.4Gy, *p* < 0.001). In a univariate analysis, there was a significant difference in the death rate between patients who received EBRT and those who did not (27% vs. 36%, OR 2.2, 95%-CI 1.1-4.3, *p* = 0.02). As mentioned in the surgery section, there was no significant difference in the mortality rate between those patients with surgery plus EBRT and those with surgery only (**Table 4**).

**Table 4:** Radiotherapy.

Total number of patients n = 176		
No EBRT	83 (47%)
EBRT with surgery	71 (40%)
EBRT without surgery	22 (13%)
Median dose (range)	41.4Gy (5 - 60Gy, SD 9.0)
Median dose EBRT with surgery	41.4 (SD 9.2)	*p* < 0.001*
Median dose EBRT without surgery	50.4 (SD 7.1)	

*Whitney-Mann U test.

### Chemotherapy

NAC was applied in 90 (51%) and AC in 75 (42%) patients. VAC (vincristine, dactinomycin, and cyclophosphamide) was the most applied regimen (47 cases, 27%) among the 176 patients. It was first used in the treatment of BRMS in the IRS Study I ^5^, that started in 1972, and has still been employed as standard chemotherapy for patients with IRS group ≥II in the most recent COG studies (1). Eleven patients did not receive any kind of chemotherapy. In five cases, the reason was a rapid disease progression with an insufficient general condition. In the other cases, the reasons are unknown. Mortality in this group was 73% (8/11). Standardized reports of chemotherapy-induced complications were lacking in most studies. However, severe complications of chemotherapy were explicitly reported in 18 cases (10%), 13 of which were fatal (7%) (**Table 5**).

**Table 5:** Chemotherapy.

Total number of patients n = 176	
No chemotherapy	11 (6%)
Neoadjuvant chemotherapy*	90 (51%)
Adjuvant chemotherapy*	75 (42%)
Severe chemotherapy-induced complications	
All reported complications	18 (10%)
Neutropenia with sepsis	13 (10%)
VOD/SOS^†^	2 (1%)
Fanconi syndrome (25)	2 (1%)
Cardiomyopathy	1 (1%)
Chemotherapy-related death	13 (7%)
Applied regimen^‡^	
VAC	47 (27%)
IVA	32 (18%)
VAIA	16 (9%)
CEVAIE	12 (7%)
VACD	12 (7%)
VAC-IE	9 (5%)
VA	7 (4%)
IVADo	5 (3%)
EVAIA	3 (2%)
Other/unknown	22 (13%)

*Definitions in the “Methods/Definitions” section. ^†^VOD, veno-occlusive disease, SOS, sinusoidal obstruction syndrome. ^‡^List of chemotherapy regimen and applied agents in **Supplementary Information 6**.

### Outcomes

#### Follow-Up

Mean and median follow-up time was 50 and 31 months, respectively (IQR 10-51). 112 children (64%) have undergone successful treatment with no evidence of disease at the time of the last follow-up. Three patients (2%) had a stable disease at the last follow-up. A total of 61 (35%) DRDs occurred. Complete remission at any point during therapy was achieved in 116 patients (66%), partial remission in 19 (11%). Disease progression during the initial therapy was observed in 37 children (21%). Of the 19 patients with IRS Group IV disease at diagnosis, six survived (32%).

#### Relapses

54 (31%) patients suffered from tumor relapse, with local relapse in 48 (27%) and distant relapse in 18 (10%). 12 patients (7%) suffered from both local and distant recurrence. The mortality rate in the 54 patients with relapse was 81% (44 deaths). Relapse mostly occurred within the first year after diagnosis (Median 6 months, range 1 – 30 months). As mentioned above, not performing tumor resection was significantly associated with relapse. There was no significant association between absence of EBRT and relapse (OR 1.6, 95%-CI 0.8-3.3, *p* = 0.14).

### Individual Patient Data Analysis

Complete individual patient data was accessible for 116 patients for the IPD analyses. 5-year OS was 51% (95% CI 42-63%) and 5-year PFS was 50% (95% CI 41-63%). For patients treated after 2000, 5-year OS was 65% compared to 54% for patients treated between 1981 – 1999, and 31% for patient treated until 1980 (*p* = 0.011). Regarding the different therapeutic regimens, not performing surgical tumor resection was significantly associated with lower survival probability compared to limited or extended surgery (*p* < 0.001). In fact, patients undergoing extended surgery had better survival than those undergoing limited surgery. Patients without surgical tumor resection had a significantly lower survival probability. Patients suffering from relapse had a significantly lower 5-year OS (8% vs. 79%, *p* = < 0.001) (**Table 6**).

**Table 6:** Patient outcome and IPD analysis.

	Total number of patients, n = 176	Treated 1952 – 1980, n = 31	Treated 1981 – 1999, n = 58	Treated after 2000, n = 87
Disease progression during initial therapy	37 (21%)	16 (52%)	11 (19%)	10 (11%)
Complete remission*	116 (66%)	13 (42%)	37 (64%)	66 (76%)
Partial remission*	19 (11%)	3 (10%)	8 (14%)	8 (9%)
Relapse*	54 (31%)	17 (55%)	18 (31%)	19 (22%)
Local relapse	48 (27%)	17 (55%)	14 (24%)	17 (20%)
Distant relapse	18 (10%)	8 (26%)	7 (12%)	3 (3%)
Disease-related death	61 (35%)	19 (61%)	21 (36%)	21 (24%)
IPD Analysis				
Mean/Median follow-up time	50/31 months (IQR 10-51)	67/46 months (IQR 5.5-29)	60/30 months (IQR 4.5-65)	34/24 months (IQR 10-36)
Median time between remission and relapse	6 months (range 1 - 30 months)	4 months (range 1 - 30 months)	12 months (range 1 - 18 months)	12 months (range 2 - 24 months)
5-year OS	51% (95% CI 41.6-63.2)	31% (95% CI 17%-55%)	54% (95% CI 40%-73%)	65% (95% CI 49%-89%)
5-year PFS	50% (95% CI 42.3-63.6)	32% (95% CI 18%-58%)	54% (95% CI 42%-74%)	59% (95% CI 40%-87%)

*Definitions in the “Methods/Definitions” section.

**Figure 2:**
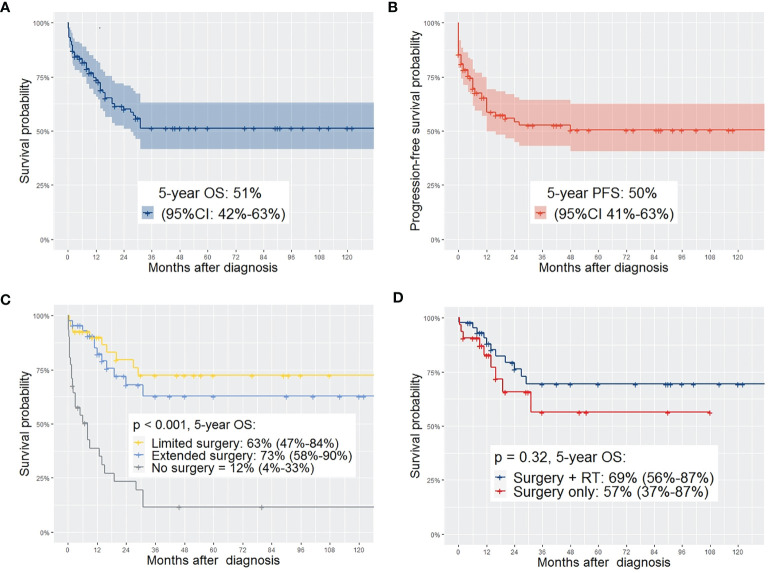
**(A)** OS of the complete study population; **(B)** PFS of the complete study population; **(C)** comparing OS depending on the type of surgery showing significant higher OS in patients with limited or extended surgery compared to no surgery (95%-CI); **(D)** comparing OS of patients with surgery plus EBRT with those who received surgery without EBRT – no significant difference in OS between the two groups (95%-CI).

In univariate analysis with the log rank test, the following factors showed significant differences (p < 0.05) in 5-year OS and/or 5-year PFS and were included as covariates in the multivariate cox regression model: treatment period, treatment within oncological study, age at diagnosis (≤ 10 or > 10 years), tumor size (≤ 5cm, > 5cm), IRS Group, histology (embryonal/botryoid/alveolar), surgical approach (limited/extended/no surgery), upfront surgery (yes/no), chemo-therapy (neoadjuvant/adjuvant/none), radiotherapy (with surgery/without surgery/none) (**Figures 2A**–**D**).

**Figure 3:**
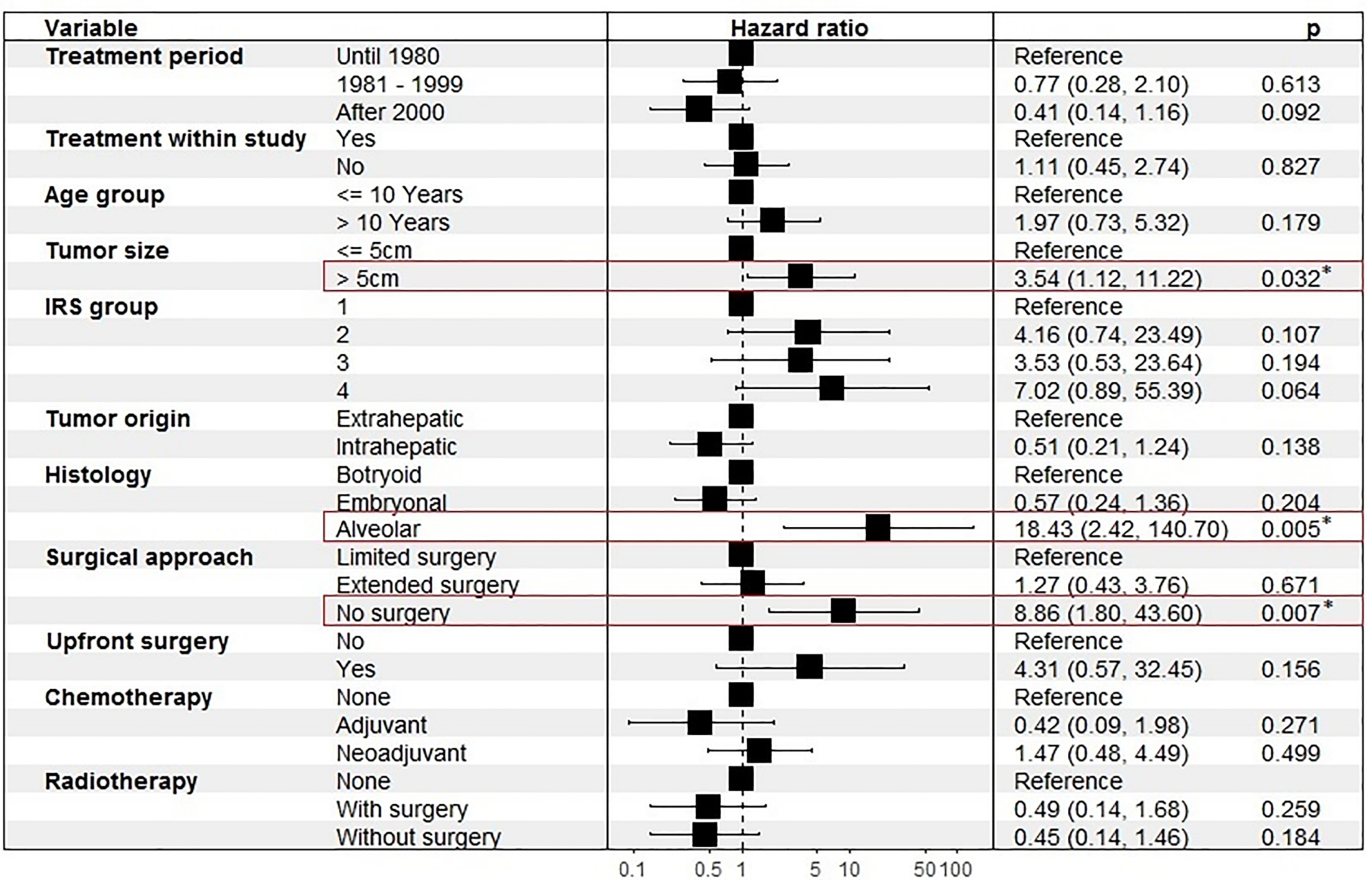
Cox proportional hazards model for risk of death, adjusted for 11 covariates. Numbers in paratheses represent 95%-CI. * marks significant results.

In a multivariate cox regression analysis, adjusted with 11 covariates, tumor size > 5cm and alveolar histology were associated with higher risk of death. The risk of death was significantly higher when no surgical tumor resection was performed (**Figure 3**).

## Discussion

This is the first systematic review and meta-analysis investigating biliary tract rhabdomyosarcoma in children. The number of included patients is more than five times higher than the largest reported cohort so far (3), thus providing comparatively strong evidence for this very rare condition.

### Patient-Related Factors and Diagnostics

Jaundice is the most common symptom in BRMS. Given the rarity of virtually all underlying diseases that cause obstructive jaundice in children, BRMS should be included as differential diagnosis for these cases. This could facilitate earlier establishment of the correct diagnosis and avoid frequent misdiagnosis as choledochal cyst or other benign processes. ERCP is an excellent diagnostic tool in cases of unclear obstructive jaundice in pediatric patients. However, ERCP is not widely available as a standard method in infants and bears the risk of life-threatening complications (24, 26). In case of inconclusive conventional diagnostics, surgical exploration is recommended, as it allows tumor biopsy, meticulous staging of BRMS and removal of the pathology even in case of a benign cause of the obstructive jaundice. The median patient age of three years at diagnosis is in line the results of previous studies (1, 3, 5). The prognosis of patients older than 10 years is significantly worse, which has been suggested by other authors before (2, 27). We report a higher prevalence of BRMS in male children, which has been indicated by most, but not all case series before (2–5). In contrast to previous assumptions (28), we found that alveolar histology can be present in BRMS and is associated with poor outcome.

### Improvements of Outcome Over Time

Our results show a tendency towards an improved treatment with better survival over the observation period. However, in multivariable analyses, the treatment period was not independently associated with a higher risk of death. In many studies, the applied chemotherapy regimens largely have consisted of the same agents for over 40 years until the present (1, 5). 5-year OS was 65% for patients treated after 2000 in our analyses and Spunt et al. have already reported a 5-year OS of 66% when analyzing patients treated in IRS studies between 1972 and 1998. The current results show that there is still a considerable mortality rate in pediatric patients with BRMS. Compared to the advantages achieved by multi-disciplinary oncological treatment in adults, an evidence-based standardization of therapy for rare tumor subtypes remains a desideratum, and joint international trials for patients with rare oncological diseases like BRMS are required.

### The Role of Surgery

A major focus of this study was to investigate the role of surgery in the therapy of BRMS, which has been discussed controversially in recent years (1–3, 5, 29). Our results suggest that surgical tumor resection should be a mainstay in the treatment of BRMS in children. This recommendation is contrasted by conclusions in recent analyses of patients with BRMS by the Children’s Oncology Group (COG) trial registries (1, 5). Other authors emphasize the need of local therapy to prevent fatal relapse and underline the importance of state-of-the art oncologic surgery (2, 3). In their study of 25 patients with BRMS treated between 1972-1998 in IRS studies I-IV, Spunt et al. concluded that extended surgery should be generally abandoned as a treatment option and that some patients with BRMS might not even benefit from any kind of surgical tumor resection (5). This opinion was reiterated in the most recent study by the COG on patients with BRMS (1). However, the authors reported a R0 resection rate of 13% and 18%, respectively (R1 25% and 18%), in all surgeries with tumor resection (1, 5), which may be viewed as subpar. In a study of the European Paediatric Soft Tissue Sarcoma Study Group (EPSSG), Guèrin et al. reported a R0 resection rate of 54% in 24 surgeries for BRMS (3). In our meta-analysis, R0 was achieved in 41% and R1 in 38% of the 128 patients who underwent operations with intent of curative tumor resection. In fact, in a univariate analysis, patients undergoing extended surgery had higher 5-year OS than those undergoing limited surgery and far higher than those without any surgical tumor resection. It is noteworthy that gross total resection with clear margins was significantly more frequent when DPR was performed after NAC. Absence of surgical tumor resection was significantly associated with DRD and relapse. Most importantly, not performing surgical therapy proofed to be an independent risk factor for death.

Without doubt, indication for surgery should always be considered carefully, and only in a context of a multimodal treatment, where chemotherapy is an indispensable and important pillar in pediatric RMS. However, our results cannot confirm the conclusions regarding the impact of surgery in BRMS drawn by previous studies with smaller caseloads (1, 5). On the contrary, surgery with intent of tumor resection should be evaluated for all patients with BRMS. On the other hand, complete remission and long-term survival without any surgical tumor resection has been reported for BRMS (4, 5, 30). Predictors or risk factors that allow for an identification of those patients who do not need surgery have not been determined yet (28, 31). Given the disastrous survival rate of patients with relapse and the association between relapse and absence of surgery in our analysis, explorative laparotomy, and tumor resection if necessary, after chemotherapy, seems advisable, also in patients with good response on imaging. This recommendation is supported by a recent study of Lautz et al., where the importance of delayed surgical tumor resection was shown for pediatric patients suffering from RMS with different primary tumor sites (13). Moreover, 5-year OS was higher in the most recent EPSSG study on BRMS (3), where 80% of the patients (24 of 30) underwent surgery, compared to survival rates in recent case series with lower surgical resection rates (1, 2).

There are aspects of surgery for BRMS which are considered problematic by some oncologists. Among these is the close relation to the vascular structures of the porta hepatis. However, for experienced hepatobiliary surgeons, meticulous dissection of these structures as well as vascular resection of the portal vein or hepatic artery is a standard procedure and presents no limitation for complete tumor resection (10, 11, 32). Moreover, liver resections in children who have received or are scheduled for chemotherapy are regarded as problematic by some authors, since toxicity of chemotherapeutic agents such as dactinomycin, ifosfamide, etoposide, or cyclophosphamide largely depends on liver metabolism (33). However, is has been clearly shown that this argument can be abandoned, since major hepatectomy is well-tolerated by children even after NAC and liver function recuperates sooner than in adults (34, 35). Additionally, the surgical techniques and the perioperative management of children has markedly improved over recent years (36, 37), enabling pediatric surgeons to perform extended procedures with higher patient safety (32). For children with advanced stage hepatoblastoma, extended liver resections, even with vascular reconstruction, achieved favorable outcomes with reasonable short-term and low long-term morbidity (32). The safety of hepatobiliary surgery in children is underlined by the low postoperative mortality and morbidity in the present study. Most acute surgical complications are manageable without long-term consequences or functional limitations. In line with this, relevant studies did not show a significant association between abdominal surgery in pediatric oncological patients and serious long-term sequelae affecting the hepatobiliary system (38–40). The resection of extrahepatic bile ducts with biliary enteric anastomosis, which is the most commonly required procedure for BRMS, has been applied for decades in children and proofed to be a safe surgery with good long-term outcome (41–43).

Chemotherapy is effective for tumor control and induces a good response in many cases of pediatric RMS, yet has considerable side effects in children, both short- and long-term (6, 12, 25, 44). Severe neutropenia and infections occur in about 80% of pediatric patients receiving chemotherapy for RMS and are fatal in 2 - 8% of patients (5, 44, 45). Long-term effects of chemotherapy, especially when combined with radiotherapy, include cardiac, hepatic and pulmonary diseases as well as an increased risk of second malignancies in survivors of pediatric malignancy (46–48). Not least because of the high rate of chemotherapy-related deaths in the present analysis and the discussed long-term sequelae, we endorse the ongoing efforts to reduce doses of chemotherapy in pediatric oncology (49). Future studies should focus on individualized treatment of pediatric patients and identify patient subgroups that profit from radical local treatment with dose reduction of chemotherapy (1, 12). Our results suggest that high-quality oncologic surgery is a key factor in this endeavor.

### Radiotherapy

The optimal application of EBRT with the minimally needed radiation dose for local tumor control is another critical variable in the treatment of BRMS. The current protocols of the respective study groups provide different guidelines concerning indication, timing, and dosage of EBRT (1, 3, 50). According to our findings, definitive EBRT does not seem to be the best suited local treatment for BRMS, considering the mortality of 41% of this subgroup in the examined population. Our results suggest that radiation doses can be significantly lowered when combined with surgery and that certain patient subgroups might not need radiation. Late adverse effects of RT in children have been shown by many studies (51–53). This has special implications in pediatric patients with BRMS, as unavoidable hepatotoxicity of chemotherapy is aggravated by liver radiation (33, 39, 40, 54, 55). EBRT involving the liver has been identified as independent risk factor for acute and late hepatic complications (39, 40, 55). Thus, one aim of future studies should be to investigate minimally needed radiation doses and to stratify patient subgroups for those who do need and those who do not need EBRT for local tumor control.

### The Biliary Tract – Favorable or Unfavorable Site of RMS?

There is an ongoing discussion concerning the classification of the biliary tract as *favorable* or *unfavorable* site of pediatric RMS in the relevant oncologic study groups (1, 3, 6, 56). Given the 5-year OS of 65% and a mortality of 24% in patients treated after 2000, our results may add to the recommendation of grading the biliary tract as unfavorable site (1). However, the current study does not present results of one homogenous oncological study but comprises patients treated according to different study protocols. The advantage of this analysis, however, is grouping of patients with BRMS, providing a homogeneous population group concerning this specific tumor location. In summary, inferences from details of the respective treatment regimens must be made with care.

### Limitations and Strengths

Several limitations of this systematic review should be noted. First, all included studies were non-randomized and non-comparative. Thus, only a moderate to low level of evidence was possible to achieve. However, it is the highest level of evidence for BRMS to date. Second, the observation period is long, which means that a comparison of patients treated under different protocols was made. To account for this bias, patients were grouped according to the time of therapy and the treatment period was included as covariate. A further limitation is the varying length and modality of follow-ups between the different studies. This further complicated a structured assessment of long-term sequelae of the disease or the therapy. Finally, the lack of necessary patient data in some studies that otherwise met the inclusion criteria also needs to be considered as a limitation. When the required information could not be retrieved, these studies either had to be excluded from the review or, in case of incomplete IPD, patients were lost for the IPD-analysis, resulting in a selection bias. Patients with all tumor stages were included in our analyses, with 19 patients suffering from metastatic disease at diagnosis. In other studies, these patients were excluded (1, 3). Given the poorer survival probability of these patients compared to those without distant disease at diagnosis, a comparison with the survival rates of studies that excluded these patients must be drawn cautiously.

Despite these limitations, the present systematic review and meta-analysis also offers major strengths. First, we performed an extensive systematic literature search and identified a high number of case reports with detailed patient data, along with all relevant studies of the respective oncologic trials. Second, with a total of 176 patients, we analyzed the highest number of patients with BRMS hitherto reported in the literature. The study design with a meta-analysis of individual patient data (IPD) is another strength, as it allowed us to control for many different variables, such as heterogeneity of patient characteristics among studies, and to avoid aggregation and ecological bias (57). Especially when aiming for a tailored and individualized therapy, IPD offers higher statistical power compared to conventional meta-analyses or meta-regression (57).

### Certainty of Evidence and Strength of Recommendations

The quality of evidence of this systematic review should be evaluated in the context of an extremely rare disease and the low level of existing evidence (21). The non-comparative design of all included studies limits the evidence to a low level. Consequently, the strength of recommendations based on our analyses is conditional. Nevertheless, our recommendation that surgery should be performed whenever resection is possible, especially explorative laparotomy with possible tumor resection after initial chemotherapy, should be strongly considered due to the following reasons: first, absence of surgical tumor resection was an independent risk factor for death, even when adjusted for ten covariates and it was significantly associated with relapse. Second, the potential harm of a mostly fatal relapse or tumor progression seems higher than the risk of a surgery, and third, there is no higher level of evidence existing.

## Data Availability Statement

The data supporting the conclusions of this article are available from the corresponding author upon reasonable request, without undue reservation.

## Author Contributions

JF, AM-L, MK, PG, AF, JP, PP, and KH contributed to conception and design of the study. JF, AM-L, and KH conducted the systematic literature search, study selection, and extracted the data. JF and AM-L performed the statistical analysis. JF wrote the initial draft of the manuscript. AM-L, KH, and AF wrote sections of the manuscript. All authors contributed to the article and approved the submitted version.

## Conflict of Interest

The authors declare that the research was conducted in the absence of any commercial or financial relationships that could be construed as a potential conflict of interest.

## Publisher’s Note

All claims expressed in this article are solely those of the authors and do not necessarily represent those of their affiliated organizations, or those of the publisher, the editors and the reviewers. Any product that may be evaluated in this article, or claim that may be made by its manufacturer, is not guaranteed or endorsed by the publisher.

## Abbreviations

AC, Adjuvant chemotherapy; BRMS, Biliary rhabdomyosarcoma; CI, Confidence interval; COG, Children’s Oncology Group; CT, Computed tomography; DPR, Delayed primary resection; DRD, Disease-related death; EBRT, External beam radiotherapy; EPSSG, European Paediatric Soft Tissue Sarcoma Study Group; ERCP, Endoscopic retrograde cholangiopancreatography; IPD, Individual patient data; IQR, Interquartile range; IRS, Intergroup Rhabdomyosarcoma Studygroup; MRI, Magnetic resonance imaging; NAC, Neoadjuvant chemotherapy; OR, Odds ratio; OS, Overall survival; PFS, Progression-free survival; RT, Radiotherapy; SD, Standard deviation; SOS, Sinusoidal obstruction syndrome; VOD, Veno-occlusive disease.
