# Supplementary material for: Biliary Rhabdomyosarcoma in Pediatric Patients: A Systematic Review and Meta-Analysis of Individual Patient Data
Source: Front Oncol. 2021 Sep 30;11:701400. doi: 10.3389/fonc.2021.701400 (PMC8515851; doi:10.3389/fonc.2021.701400)
Supplement: Supplementary file 1 [file DataSheet_1.zip › Supplementary_material_1.docx]

Supporting information 1: List of included studies

| Authors | Title | Year of publication | Cases of BRMS | Country |
| --- | --- | --- | --- | --- |
| Aggarwal et al. | *Botryoid Rhabdomyosarcoma of Common Bile Duct* | 2004 | 1 | India |
| Akers et al. | *Sarcoma Botryoides (Rhabdomyosarcoma) of the Bile Ducts With Survival* | 1971 | 1 | USA |
| Alhashem et al. | *Biliary Rhabdomyosarcoma in an Infant Male With Neurofibromatosis Type 1* | 2019 | 1 | Turkey |
| AlQuran et al. | *Laparoscopic management of Rhabdomyosarcoma of common Bile duct, Case report* | 2020 | 1 | Jordan |
| Arnaud et al. | *Embryonal rhabdomyosarcoma of the biliary tree in children: a case report* | 1987 | 1 | France |
| Aye et al. | *Suboptimal outcome for patients with biliary rhabdomyosarcoma treated on low-risk clinical trials: A report from the Children’s Oncology Group* | 2021 | 17 | USA |
| Bässler et al. | *Pathologie und submikroskopische Morphologie des sogenannten Sarcoma botryoides der groBen Gallengäinge* | 1962 | 1 | Germany |
| Bar-Maor et al. | *Rhabdomyosarcoma of the common bile duct imitating choledochal cyst* | 1989 | 1 | Israel |
| Chowdhary et al. | *Rhabdomyosarcoma of Biliary Tract Misdiagnosed as Choledochal Cyst-Two Cases and Review of Literature* | 2016 | 2 | India |
| Davis et al. | *EMBONAL RHABDOMYOSARCOMA (SARCOMA BOTRYOIDES) OF THE BILIARY TREE* | 1969 | 5 | USA |
| D'Ambrosio et al. | *Pancreaticoduodenectomy for malignancies in children* | 2013 | 2 | Italy |
| Delany et al. | *Sarcoma Botryoides of the Common Bile Duct - Report of a Case and Review of the Literature* | 1969 | 1 | USA |
| Farinacci et al. | *SARCOMA BOTRYOIDES (A FORM OF EMBRYONAL RHABDOMYOSARCOMA) OF THE COMMON BILE DUCT* | 1956 | 2 | USA |
| Friedburg et al. | *Sonographic and computed tomographic features of embryonal rhabdomyosarcoma of the biliary tract* | 1984 | 1 | Germany |
| Gonzalez et al. | *Rabdomiosarcoma Botroides de vías biliares - A propósito de un caso y revisión de la literatura* | 2006 | 1 | Mexico |
| Guèrin et al. | *Outcome of localized liver-bile duct rhabdomyosarcoma according to local therapy: A report from the European*  *Paediatric Soft-Tissue Sarcoma Study Group (EpSSG)-RMS 2005 study* | 2019 | 30 | France (international study group) |
| Gururangan et al. | *Primary Hepatic Tumours in Children:*  *A 2-Year Review* | 1992 | 2 | Ireland |
| Haider et al. | *Primary Embryonal Rhabdomyosarcoma*  *of the Liver in a Young Male* | 2013 | 1 | India |
| Himes et al. | *Diagnostic and therapeutic role of endoscopic retrograde cholangiopancreatography in biliary rhabdomyosarcoma* | 2008 | 1 | USA |
| Horn et al. | *RHABDOMYOSARCOMA (SARCOMA BOTRYOIDES) OF THE COMMON BILE DUCT* | 1954 | 1 | USA |
| Horrowitz et al. | *Hepatic Undifferentiated (Embryonal) Sarcoma and Rhabdomyosarcoma In Children* | 1987 | 3 | USA |
| Huber et al. | *Long Term Survival in two Children with*  *Rhabdomyosarcoma of the Biliary Tract* | 2008 | 2 | Austria |
| Isaacson et al. | *EMBRYONAL RHABDOMYOSARCOMA OF THE AMPULLA OF VATER* | 1978 | 1 | South Africa |
| Kebudi et al. | *Rhabdomyosarcoma of the biliary tree* | 2003 | 1 | Turkey |
| Kirli et al. | *Rhabdomyosarcoma of the common bile duct: an unusual cause of obstructive jaundice in a child* | 2012 | 1 | Turkey |
| Kitagawa et al | *Biliary rhabdomyosarcoma* | 2007 | 1 | Japan |
| Kouadir et al. | *Rhabdomyosarcoma of the biliary tract: a case report* | 2017 | 1 | Morocco |
| Kumar et al. | *Rhabdomyosarcoma of Biliary Tract—a Diagnostic Dilemma* | 2012 | 1 | India |
| Lack et al. | *Botryoid rhabdomyosarcoma of the biliary tract* | 1981 | 5 | USA |
| Majmudar et al. | *EMBRYONAL RHABDOMYOSARCOMA*  *(SARCOMA BOTRYOIDES) OF*  *THE COMMON BILE DUCT:*  *A CASE REPORT* | 1976 | 1 | USA |
| Margain et al. | *A Botryoid Rhabdomyosarcoma Diagnosed as a Choledochal Cyst* | 2013 | 1 | France |
| Martinez et al. | *Rhabdomyosarcoma of the Biliary Tree:*  *The Case for Aggressive Surgery* | 1982 | 3 | USA |
| Mathew et al. | *Embryonal rhabdomyosarcoma of the biliary tree in a paediatric patient – A rare cause of obstructive jaundice* | 2019 | 1 | South Africa |
| McCannon et al. | *The use of percutaneous transhepatic cholangiography in a case of embryonal rhabdomyosarcoma* | 1978 | 1 | Ireland |
| Mihara et al. | *Botryoid Rhabdomyosarcoma of the Gallbladder in a Child* | 1982 | 1 | Japan |
| Nagaraj et al. | *Rhabdomyosarcoma of the Bile Ducts* | 1977 | 1 | USA |
| Nakib et al. | *Robotic-assisted surgery approach in a biliary rhabdomyosarcoma misdiagnosed as choledochal cyst* | 2014 | 1 | Italy |
| Noskiewicz et al. | *Huge Liver Tumor - Therapy Outside the Guidelines* | 2018 | 1 | Poland |
| Oelsnitz et al. | *Embryonal Rhabdomyosarcoma of the Common Bile Duct* | 1991 | 1 | Germany |
| Paganelli | *A child with unresectable biliary*  *rhabdomyosarcoma: 48-month disease-free survival after liver transplantation* | 2014 | 1 | Italy |
| Patel et al. | *Potential alternative treatment approach for pediatric patient with diffusely infiltrative primary rhabdomyosarcoma of the liver* | 2021 | 1 | USA |
| Pater et al. | *Pencil Beam Scanning Proton Therapy for Rhabdomyosarcoma of the Biliary Tract* | 2017 | 1 | USA |
| Perera et al. | *Embryonal rhabdomyosarcoma of the ampulla of Vater in early childhood: report of a case and review of literature* | 2008 | 1 | United Kingdom |
| Perirsic et al. | *Cholestasis Caused by Biliary Botryoid Sarcoma* | 1991 | 1 | Serbia |
| Perruccio et al. | *Biliary tract rhabdomyosarcoma: a report from the Soft Tissue Sarcoma Committee of the Associazione Italiana Ematologia Oncologia Pediatrica* | 2018 | 10 | Italy |
| Pollono et al. | *Rhabdomyosarcoma of Extrahepatic Biliary Tree: Initial Treatment With*  *Chemotherapy and Conservative Surgery* | 1998 | 1 | Argentina |
| Prasad et al. | *Embryonal Rhabdomyosarcoma of the Common Bile Duct* | 2003 | 1 | India |
| Rajendran et al. | *Embryonal rhabdomyosarcoma of liver in a 16-year-old boy: A rare case report* | 2014 | 1 | India |
| Roebuck et al. | *Hepatobiliary rhabdomyosarcoma*  *in children: diagnostic radiology* | 1998 | 4 | China |
| Rojas et al. | *Rhabdomyosarcoma arising within the biliary tract mimicking a choledochal cyst: A case report* | 2016 | 1 | USA |
| Sanz et al. | *Rhabdomyosarcoma of the biliary tree* | 1997 | 1 | Spain |
| Sassi et al. | *Une choléstase causée par un rhabdomyosarcoma botryoïde du cholédoque chez un nourrisson de 22 mois* | 2008 | 1 | Tunisia |
| Schweitzer et al. | *Major resection for embryonal rhabdomyosarcoma of the biliary tree* | 1994 | 4 | Germany |
| Scottoni et al. | *ERCP with intracholedocal biopsy for the diagnosis of biliary tract rhabdomyosarcoma in children* | 2013 | 2 | Italy |
| Shen et al. | *Liver Transplantation for Biliary Rhabdomyosarcoma With Liver*  *Metastasis: Report of One Case* | 2016 | 1 | China |
| Spunt et al. | *Aggressive Surgery Is Unwarranted for Biliary Tract Rhabdomyosarcoma* | 2000 | 25 | USA |
| Taira et al. | *SARCOMA BOTRYOIDES ARISING FROM THE BILIARY TRACT OF THE LITERATURE OF CHILDREN - A CASE REPORT WITH REVIEW* | 1976 | 1 | Japan |
| Tireli et al. | *Embryonal rhabdomyosarcoma of the common bile duct mimicking choledochal cyst* | 2005 | 1 | Turkey |
| Tsuchiya et al. | *Malignant Tumors in Choledochal Cysts* | 1977 | 1 | Japan |
| Tugcu et al. | *Rhabdomyosarcoma of the Common Bile Duct: Case Report* | 2005 | 1 | Turkey |
| Upadhyaya et al. | *Primary hepatic sarcomas in children—a single-center experience over 19 years* | 2010 | 6 | United Kingdom |
| Urla et al. | *Treatment and outcome of the patients with rhabdomyosarcoma of the biliary tree: Experience of the Cooperative Weichteilsarkom Studiengruppe (CWS)* | 2019 | 17 | Germany (international study group) |
| Verstandig et al. | *Sarcoma botryoides of the common bile duct: preoperative diagnosis by coronal CT and PTC* | 1990 | 1 | Israel |
| Williams et al. | *Ultrasound Appearance of Biliary*  *Rhabdomyosarcoma* | 1986 | 1 | USA |
| Zampieri et al. | *Botryoid rhabdomyosarcoma of the biliary tract in children: a unique case report* | 2006 | 1 | Italy |
