# Supplementary material for: Biliary Rhabdomyosarcoma in Pediatric Patients: A Systematic Review and Meta-Analysis of Individual Patient Data
Source: Front Oncol. 2021 Sep 30;11:701400. doi: 10.3389/fonc.2021.701400 (PMC8515851; doi:10.3389/fonc.2021.701400)
Supplement: Supplementary file 1 [file DataSheet_1.zip › Supplementary_material_2.docx]

Supporting information 2: Risk of bias assessment of case reports/series

| Study | Selection | Ascertainment | Causality | Reporting | Overall risk of bias |
| --- | --- | --- | --- | --- | --- |
| Aggarwal et al. | high | moderate | moderate | high | high |
| Akers et al. | high | low | low | low | moderate |
| Alhashem et al. | high | low | low | moderate | moderate |
| AlQuran et al. | high | moderate | moderate | high | high |
| Arnaud et al. | high | moderate | high | moderate | high |
| Bässler et al. | high | moderate | high | high | high |
| Bar-Maor et al. | high | moderate | moderate | moderate | moderate |
| Chowdhary et al. | high | moderate | moderate | high | high |
| Davis et al. | low | moderate | moderate | high | high |
| D'Ambrosio et al. | moderate | moderate | moderate | moderate | moderate |
| Delany et al. | high | moderate | moderate | moderate | moderate |
| Farinacci et al. | high | moderate | moderate | moderate | moderate |
| Friedburg et al. | high | low | moderate | high | moderate |
| Gonzalez et al. | high | moderate | moderate | moderate | moderate |
| Gururangan et al. | low | moderate | moderate | moderate | moderate |
| Haider et al. | high | moderate | low | moderate | moderate |
| Himes et al. | high | low | high | moderate | high |
| Horn et al. | high | moderate | high | low | high |
| Horrowitz et al. | very low | low | low | moderate | high |
| Huber et al. | moderate | moderate | low | moderate | low |
| Isaacson et al. | high | moderate | high | moderate | moderate |
| Kebudi et al. | high | moderate | moderate | moderate | moderate |
| Kirli et al. | high | moderate | moderate | moderate | moderate |
| Kitagawa et al | high | high | high | high | high |
| Kouadir et al. | high | moderate | moderate | moderate | moderate |
| Kumar et al. | high | moderate | high | moderate | high |
| Lack et al. | very low | low | moderate | high | high |
| Majmudar et al. | high | moderate | moderate | moderate | moderate |
| Margain et al. | high | moderate | moderate | high | moderate |
| Martinez et al. | high | moderate | moderate | moderate | moderate |
| Mathew et al. | high | moderate | high | moderate | moderate |
| McCannon et al. | high | moderate | moderate | moderate | moderate |
| Mihara et al. | high | low | moderate | low | low |
| Nagaraj et al. | high | low | high | moderate | moderate |
| Nakib et al. | high | moderate | moderate | moderate | moderate |
| Noskiewicz et al. | high | low | low | moderate | low |
| Oelsnitz et al. | high | low | low | low | low |
| Paganelli | moderate | low | low | low | low |
| Patel et al. | high | moderate | low | moderate | low |
| Pater et al. | high | moderate | moderate | moderate | moderate |
| Perera et al. | high | moderate | moderate | moderate | moderate |
| Perirsic et al. | high | low | moderate | moderate | moderate |
| Pollono et al. | high | low | low | moderate | low |
| Prasad et al. | high | moderate | moderate | moderate | moderate |
| Rajendran et al. | high | moderate | moderate | moderate | moderate |
| Roebuck et al. | moderate | moderate | moderate | moderate | moderate |
| Rojas et al. | high | moderate | moderate | moderate | moderate |
| Sanz et al. | high | high | high | moderate | high |
| Sassi et al. | high | moderate | moderate | low | moderate |
| Schweitzer et al. | moderate | moderate | moderate | low | low |
| Scottoni et al. | moderate | low | moderate | moderate | moderate |
| Shen et al. | high | low | moderate | moderate | moderate |
| Taira et al. | high | moderate | moderate | high | high |
| Tireli et al. | high | moderate | moderate | high | high |
| Tsuchiya et al. | low | low | moderate | moderate | moderate |
| Tugcu et al. | high | high | moderate | high | high |
| Upadhyaya et al. | low | moderate | moderate | high | high |
| Verstandig et al. | high | moderate | high | moderate | moderate |
| Williams et al. | high | moderate | high | moderate | high |
| Zampieri et al. | high | low | low | low | low |
