# Supplementary material for: Biliary Rhabdomyosarcoma in Pediatric Patients: A Systematic Review and Meta-Analysis of Individual Patient Data
Source: Front Oncol. 2021 Sep 30;11:701400. doi: 10.3389/fonc.2021.701400 (PMC8515851; doi:10.3389/fonc.2021.701400)
Supplement: Supplementary file 1 [file DataSheet_1.zip › Supplementary_material_3.docx]

Supporting information 3: Risk of bias of observational studies (MINORS)

| MINORS Items | Spunt et al. | Perruccio et al. | Urla et al. | Guèrin et al | Aye et al. |
| --- | --- | --- | --- | --- | --- |
| Clearly stated aim | 2 | 1 | 2 | 2 | 1 |
| Inclusion of consecutive patients | 2 | 2 | 2 | 2 | 2 |
| Prospective collection of data | 2 | 2 | 2 | 2 | 2 |
| Endpoints appropriate to aim of study | 2 | 1 | 2 | 2 | 2 |
| Unbiased assessment of the study endpoint | 2 | 2 | 2 | 2 | 2 |
| Follow-up period appropriate to aim of study | 1 | 2 | 2 | 2 | 2 |
| Loss to follow up less than 5% | 2 | 2 | 2 | 2 | 2 |
| Prospective calculation of the study size | 0 | 0 | 0 | 0 | 0 |
| Total | 14/16 | 13/16 | 14/16 | 14/16 | 13/16 |
