# Supplementary material for: Biliary Rhabdomyosarcoma in Pediatric Patients: A Systematic Review and Meta-Analysis of Individual Patient Data
Source: Front Oncol. 2021 Sep 30;11:701400. doi: 10.3389/fonc.2021.701400 (PMC8515851; doi:10.3389/fonc.2021.701400)
Supplement: Supplementary file 1 [file DataSheet_1.zip › Supplementary_material_4.docx]

Supporting information 4: List of collected variables:

| Author names | Tumor size | Local radiotherapy |
| --- | --- | --- |
| Article title | T status | Radiotherapy details |
| Year of publication | N status | Timing of radiotherapy |
| Country | M status | Radiotherapy +/- surgery |
| Number of patients reported | IRS group | Disease progression during initial therapy |
| Treatment within study | Initial treatment | Complete remission |
| Year of diagnosis | Biopsy (method) | Partial remission |
| Treatment period | External biliary drainage | Relapse |
| Age at diagnosis | Chemotherapy (neoadjuvant/adjuvant/none) | Local relapse |
| Gender | Chemotherapy regimen | Distant relapse |
| Comorbidities | Chemotherapy complications | Time from remission to relapse |
| Initially suspected diagnosis | Response | Outcome |
| Presenting symptoms | Up-front surgery | DRD |
| Duration of symptoms | Delayed primary resection | Time from diagnosis to DRD |
| Bilirubin at diagnosis[mg/dl] | Re-resection | Cause of death |
| X-ray findings | Name of surgical procedure | Last follow-up |
| Ultrasound findings | Surgical procedure type | Overall survival |
| ERCP findings | Surgical approach | Disease-free survival |
| CT findings | R-status (first surgery) | Progression-free survival |
| MRI findings | R-status (delayed/second surgery) | Late sequelae |
| Additional diagnostics | Postoperative complications | Additional remarks |
| Tumor origin | Reoperations |  |
| Tumor location | Residual disease after local treatment |  |
