# Supplementary material for: Biliary Rhabdomyosarcoma in Pediatric Patients: A Systematic Review and Meta-Analysis of Individual Patient Data
Source: Front Oncol. 2021 Sep 30;11:701400. doi: 10.3389/fonc.2021.701400 (PMC8515851; doi:10.3389/fonc.2021.701400)
Supplement: Supplementary file 1 [file DataSheet_1.zip › Supplementary_material_5.docx]

Supporting information 5: List of excluded studies due to lacking information/data

| Authors | Title | Year of publication | Cases of BRMS | Country |
| --- | --- | --- | --- | --- |
| Hays et al. | *BOTRYOID SARCOMA (RHABDOMYOSARCOMA)*  *OF THE BILE DUCTS* | 1965 | 2 | USA |
| Soper et al. | *SARCOMA BOTFIYOIDES OF THE BILIARY TREE* | 1968 | 1 | USA |
| Taura et al. | *ULTRASTRUCTURE OF BOTRYOID SARCOMA*  *OF THE COMMON BILE DUCT* | 1977 | 1 | Japan |
| Geoffray et al. | *Ultrasonography and computed tomography for diagnosis and follow-up of biliary duct rhabdomyosarcomas in children* | 1987 | 8 | France |
| Sharma et al. | *Obstructive jaundice in children may be due to a malignant tumour*  *of the common bile duct* | 1995 | 1 | India |
| Tutar et al. | *Primary embryonal botryoid-type rhabdomyosarcoma of the liver* | 2007 | 1 | Turkey |
| Edil et al. | *Choledochal Cyst Disease in Children and Adults:*  *A 30-Year Single-Institution Experience* | 2008 | 1 | USA |
| Ali et al. | *Biliary Rhabdomyoscarcoma Mimicking Choledochal Cyst* | 2009 | 1 | USA |
| Skollik et al. | *Jaundice, pruritus, and biliary dilatation* | 2009 | 1 | United Kingdom |
| Lad eta al. | *Botyroid Rhabdomyosarcoma of the Common Bile Duct* | 2010 | 1 | India |
| Zama et al. | *Hepatic tumours in childhood: An experience at the Children Hospital and Institute of Child Health, Lahore* | 2011 | 1 | India |
| Diaconescu et al. | *Childhood rhabdomyosarcoma. Anatomo-clinical*  *and therapeutic study on 25 cases. Surgical implications* | 2013 | 1 | Romania |
| Daram et al. | *Obstructive jaundice in a child: usual presentation of an unusual tumor* | 2014 | 1 | USA |
| Elwahab et al. | *Hepatobiliary rhabdomyosarcoma mimicking choledochal cyst: Lessons learned* | 2014 | 1 | Egypt |
| Ugras et al. | *Embryonal rhabdomyosarcoma of the common bile duct and porta hepatis in an infant: A rare presentation* | 2014 | 1 | Turkey |
| Raina et al. | *Botryoid Rhabdomyosarcoma of Biliary Tree: A Diagnostic Puzzle* | 2015 | 1 | India |
| Kinariwala et al. | *Embryonal rhabdomyosarcoma of the biliary tree: A rare cause of obstructive jaundice in children which can mimic choledochal cysts* | 2017 | 1 | USA |
| Farkas et al. | *Application of ERCP and Spyglass Technique in Diagnosis of PediatricvBotryoid Rhabdomyosarcoma of the Biliary Duct* | 2019 | 1 | USA |
| Borges et al. | *A Rare Association With Obstructive Jaundice* | 2020 | 1 | Portugal |
