# Supplementary material for: Biliary Rhabdomyosarcoma in Pediatric Patients: A Systematic Review and Meta-Analysis of Individual Patient Data
Source: Front Oncol. 2021 Sep 30;11:701400. doi: 10.3389/fonc.2021.701400 (PMC8515851; doi:10.3389/fonc.2021.701400)
Supplement: Supplementary file 1 [file DataSheet_1.zip › Supplementary_material_6.docx]

Supporting information 6: Chemotherapy regimen

| Applied chemotherapeutic regimen in the included studies | |
| --- | --- |
| VAC | Vincristine, Actinomycin-d, Cyclophosphamide |
| IVA | Ifosfamide, Vincristine, Actinomycin-d |
| VAIA | Vincristine, Doxorubicin (Adriamycin), Ifosfamide, Acintomycin-d |
| CEVAIE | Carboplatin, Epirubicin, Vincristine, Ifosfamide, Actinomycin-d, Etoposide |
| VACD | Vincristine, Actinomycin-d, Cyclophosphamide, Doxorubicin |
| VAC-IE | Vincristine, Actinomycin-d, Cyclophosphamide, Ifosfamide, Etoposide |
| VA | Vincristine, Actinomycin-d |
| IVADo | Ifosfamide, Vincristine, Actinomycin-d, Doxorubicin |
| EVAIA | Etoposide, Vincristine, Doxorubicin, Ifosfamide, Actinomycin-d |
