# Supplementary material for: Biliary Rhabdomyosarcoma in Pediatric Patients: A Systematic Review and Meta-Analysis of Individual Patient Data
Source: Front Oncol. 2021 Sep 30;11:701400. doi: 10.3389/fonc.2021.701400 (PMC8515851; doi:10.3389/fonc.2021.701400)
Supplement: Supplementary file 1 [file DataSheet_1.zip › Supplementary_material_7.docx]

Supporting information 7: Applied search strategies

Medline (via Pubmed): ((biliary[tiab] OR hepatobiliary[tiab] OR botryoides[tiab] OR bile duct[tiab] or botryoid) AND (rhabdomyosarcoma*[tiab] OR sarcoma*[tiab])) OR ((Bile duct neoplasms[MeSh] or Biliary Tract Neoplasms[MeSh] or Botryoid rhabdomyosarcoma[MeSh]) AND Child[MeSH])

Web of Science: TS = ((biliary OR bile duct OR liver) AND (rhabdomyosarcoma* OR sarcoma*) AND (child or children or pediatric or infant* or adolescent*) )

Central: ((biliary or bile duct)):ti,ab,kw AND ((rhabdomyosarcoma or sarcoma)):ti,ab,kw
